# Mechanistic pathways predictive modeling and translational interventions for radiation enteritis in cervical cancer radiotherapy

**DOI:** 10.3389/fcimb.2025.1729528

**Published:** 2026-01-29

**Authors:** Xiaodong Wang, Di Xiong, Bingchen Duan, Yiping Huang, Gouping Ding, Yixuan Tang, Qianqian Wang

**Affiliations:** 1 Department of Oncology, Zhuzhou Hospital Affiliated to Xiangya School of Medicine, Central South University, Zhuzhou, China; 2 Department of General Medicine, Zhuzhou Hospital Affiliated to Xiangya School of Medicine, Central South University, Zhuzhou, China; 3 Department of Orthopaedic Surgery, Zhuzhou Hospital Affiliated to Xiangya School of Medicine, Central South University, Zhuzhou, China

**Keywords:** cervical cancer, DNA damage response, predictive modeling, radiation enteritis, translational therapeutics

## Abstract

Radiation enteritis remains a major dose-limiting toxicity in cervical cancer radiotherapy, significantly impairing treatment continuity, long-term gastrointestinal function, and patient quality of life. Despite advances in radiation techniques, the biological heterogeneity of intestinal radiosensitivity continues to challenge effective prevention and management. This review synthesizes current evidence on the core mechanistic axes underlying radiation enteritis, with a focus on DNA damage response failure, oxidative stress amplification, immune dysregulation, and microbiota disruption. We further summarize emerging predictive frameworks integrating clinical variables, dosimetric parameters, radiomics, and circulating biomarkers to enable individualized risk stratification. Particular attention is given to translational therapeutic strategies, including antioxidant pathway modulation, inflammasome targeting, microbiota engineering, and tissue-protective agents, highlighting both their mechanistic rationale and clinical feasibility. By linking molecular pathophysiology with predictive modeling and intervention development, this review provides an integrated roadmap for precision prevention and management of radiation enteritis in cervical cancer radiotherapy. Such a framework may facilitate risk-adapted treatment planning, mitigate gastrointestinal toxicity, and ultimately improve therapeutic outcomes.

## Graphical Abstract

**Figure:**
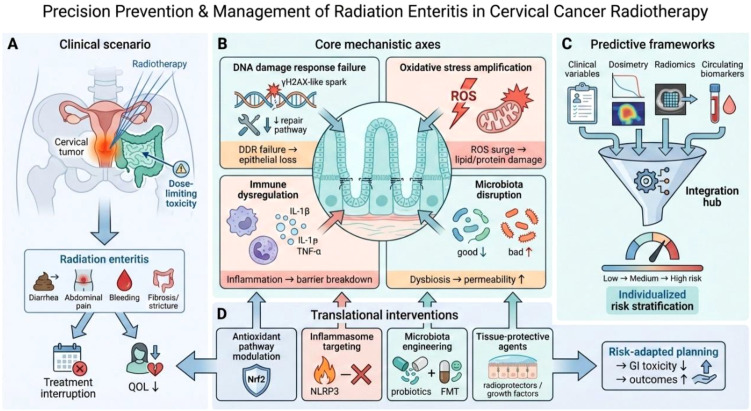

## 1 Introduction

Radiation enteritis (RE) is a frequent and clinically significant toxicity of pelvic radiotherapy for cervical cancer, particularly when combined with concurrent chemotherapy (Ma et al., 2023; Zhu et al., 2025). Ionizing radiation inevitably injures segments of the small and large intestines within the treatment field. Clinically, RE presents in acute and chronic forms (Ma C-y. et al., 2025). Acute RE arises within weeks of therapy, manifesting as diarrhea, cramping, and tenesmus driven by mucosal edema, erosion, and intense inflammatory infiltration (Ma C-y. et al., 2025). Over 60% of elderly cervical cancer patients undergoing chemoradiation develop clinically meaningful acute RE, often necessitating treatment breaks and predisposing to malnutrition and infection (Ma et al., 2023; Zhu et al., 2025). Chronic RE emerges months to years later and is defined by progressive obliterative vasculopathy, fibrosis, and compromised blood supply, resulting in strictures, malabsorption, and bleeding (Chater et al., 2019; Ma et al., 2023). Approximately 15% of severe cases ultimately require surgical intervention (Chater et al., 2019). Histologically, chronic RE comprises mixed, fibrotic, telangiectatic, and ulcerative subtypes, each associated with distinct complications – friable telangiectasias with bleeding, fistula-forming ulcerations, or obstructive fibrosis (Chater et al., 2019). As therapeutic advances prolong survival, the cumulative burden of RE on quality of life and healthcare systems continues to rise.

Beyond direct morbidity, RE constrains the safe delivery of optimal radiotherapy, imposing a “normal tissue tolerance ceiling.” (Ma et al., 2024) Its pathogenesis is multifactorial, involving interlinked processes of genomic DNA damage, oxidative stress, inflammatory–immune activation, epithelial barrier disruption, dysregulated programmed cell death, and gut microbiota dysbiosis (Ma et al., 2024; Ma C-y. et al., 2025) (**Table 1**). These mechanisms drive acute mucosal injury and, when unresolved, sustain chronic inflammation and fibrosis. Cervical cancer–specific factors further shape RE biology: fractionated dosing (typically 45–50 Gy with brachytherapy boosts) and radiosensitizing chemotherapy amplify mucosal vulnerability (You et al., 2019; Ma et al., 2024). Higher cumulative pelvic doses (≥75 Gy) correlate with more severe chronic proctitis, and concurrent chemotherapy exacerbates epithelial injury and delays healing (Mazeron et al., 2016; Deng et al., 2025). Given the prevalence in relatively young women receiving multimodality therapy, understanding RE within the cervical cancer context is essential.

**Table 1:** Key pathogenic pathways in radiation enteritis (RE) during cervical cancer radiotherapy.

Pathway	Key mechanisms/molecules	Role in RE	Clinical relevance	
DNA Damage and Repair	DSBs activating ATM/cGAS-STING; USP15–ATM stabilization; Nrf2–Pirin interaction; p53-dependent apoptosis	Initiates genomic instability, crypt cell death, and interferon-mediated inflammation; unresolved DSBs lead to villus denudation and fibrosis	Limits safe radiation dose; cervical chemoradiation produces a high DSB burden, and patients with suboptimal DNA repair (e.g., older age) have more severe acute and chronic RE
Oxidative Stress	ROS generation (·OH, O_2_^−^, H_2_O_2_); Nrf2/Keap1 activation of HO-1/NQO1/GPX4; lipid peroxidation	Causes membrane damage, mitochondrial fission, and crypt hypoplasia in acute phase; sustains myofibroblast activation and fibrosis in chronic phase	Major contributor to acute mucosal injury after pelvic RT; patients with diminished antioxidant capacity (e.g., older or comorbid) suffer worse RE, suggesting Nrf2-boosting antioxidants may mitigate toxicity
Immune-Inflammatory Response	DAMPs activating TLRs/NF-κB; NLRP3 inflammasome (IL-1β, IL-18); TH17 dominance (IL-17A, IL-22); cGAS-STING interferon production	Drives acute cytokine storm, neutrophil influx, and pyroptosis; chronic SASP (senescence-associated secretory phenotype) promotes EndoMT (endothelial-to-mesenchymal transition) and fibrosis (fibrotic/telangiectatic subtypes)	Underlies acute GI toxicity (diarrhea, pain) via cytokine surge; persistent inflammation (e.g., TH17/IL-17 axis) promotes chronic fibrosis. Immunomodulators targeting these pathways may lessen RE severity in cervical cancer RT
Epithelial Barrier Disruption	Notch/Wnt imbalance; Lgr5^+^ stem cell depletion; tight junction loss (Claudin-1, Occludin, ZO-1)	Leads to villus atrophy, bacterial translocation, and barrier failure; impairs intestinal stem cell (ISC) regeneration in acute phase, contributing to chronic malabsorption	Compromises the gut barrier during chemoradiation; allows bacterial translocation and sepsis risk in acute RE, and malabsorption in chronic RE. Strategies to preserve epithelial integrity (e.g., probiotics or growth factors) are critical to mitigate toxicity
Ferroptosis and Apoptosis	PPARγ–GAPDH axis; ACSL4-mediated lipid peroxidation; Bax/Bcl-2–Caspase-3/PARP cascade	Interplays with oxidative stress to induce programmed cell death in intestinal epithelial and endothelial cells; exacerbates acute mucosal damage and chronic ulceration	Causes massive gut cell loss; crypt apoptosis drives acute mucosal erosion, while ferroptotic endothelial injury leads to chronic ulceration. Balanced inhibition of these death pathways could improve normal tissue tolerance in cervical RT
Microbiota Dysbiosis	Depletion of short-chain fatty acid (SCFA)-producing Firmicutes (e.g., *Roseburia*, *Faecalibacterium*); increased luminal phenethylamine; indole-3-ethanol imbalance	Enhances systemic inflammation, ferroptosis, and barrier disruption; links to chronic RE via RIG-I/Notch hyperactivation	Exacerbates radiation injury; loss of beneficial SCFA-producing microbes and metabolites leads to heightened inflammation and impaired healing. Cervical cancer patients with dysbiosis have greater RE severity, suggesting microbiome restoration (probiotics/FMT) could reduce toxicity

**Table 1** summarizes the principal molecular and cellular pathways implicated in the pathogenesis of radiation enteritis, highlighting key mediators, their mechanistic roles in intestinal injury, and their relevance to clinical severity, susceptibility, and long-term outcomes.

Recent advances in predictive tools enable increasingly personalized RE risk estimation (**Table 2**). Nomogram models that integrate patient age, radiation dose–volume metrics, and comorbidities can predict ≥Grade 2 acute RE with over 80% accuracy in older cervical cancer patients (Wang and Hu, 2022; Zhu et al., 2025). Delta-radiomics MRI approaches achieve area-under-curve values above 0.90 for forecasting radiation proctitis in cervical cancer cohorts (Xue et al., 2025). Likewise, microbiome–metabolome signatures – for example, reduced Faecalibacterium prausnitzii and elevated luminal phenylethylamine – predict severe acute RE with approximately 97% specificity (Ma et al., 2024; Ma C-y. et al., 2025). These precision-medicine strategies underscore substantial inter-patient variability and support risk-adapted mitigation.

**Table 2:** Predictive models and biomarkers for severe acute and chronic RE.

Model/biomarker type	Components/features	Predictive performance (AUC/accuracy)	Applications/notes
Clinical–Dosimetric Nomogram	Age, hypertension, diabetes, mean rectal dose (Dmean_R), V20 > 370 cm³, lactate dehydrogenase-to-albumin ratio (LAR)	AUC 0.82–0.90; accuracy > 82%	Risk stratification for ≥ grade 2 acute RE in older adults; enables dose modifications
Delta-Radiomics Model*MRI/CT-based*	Serial MRI/CT features (e.g., T1_wavelet-LLL_glcm_MCC; D1cc, D2cc); Lasso regression for feature selection	AUC 0.90–0.92 (train/validation)	Predicts proctitis severity; identifies texture changes for proactive supportive care
Microbiome–Metabolome Nomogram	Fecal phenethylamine, COX-2, *Faecalibacterium prausnitzii* abundance; combined with dosimetric parameter (V25 > 290 cm³)	AUC 0.975–0.98	Noninvasive prediction of severe acute RE; supports multi-omics prognostication
Transformer-Based Deep Learning*SAM-Med2D model*	CT imaging texture features; integrated delta-radiomics (e.g., T2_original_firstorder_90 Percentile)	AUC 0.90	Identifies personalized imaging features for follow-up; aids in radiation proctitis diagnosis
Serological Biomarkers	IL-22 baseline level; CD13/CCL28/EN-RAGE (via Mendelian randomization); fecal calprotectin trends	OR 1.87 for LAR; (odds ratio) predictive for crypt radiosensitivity	Stratifies likely responders to probiotic + 5-ASA therapy; monitors early indicators of chronic RE development

**Table 2** outlines representative predictive models and biomarkers for radiation enteritis, including clinical–dosimetric nomograms, radiomics-based approaches, and biological indicators, with emphasis on their predictive performance and potential applications in individualized risk assessment.

This review delineates the core mechanistic axes of RE – DNA damage and repair responses, oxidative stress, immune-inflammatory cascades, epithelial barrier and stem-cell injury, and programmed cell-death pathways – within the specific framework of cervical cancer pelvic radiotherapy. We then summarize modulators and emerging interventions targeting these axes, including pharmacologic agents, cell-based therapies, microbiome-directed strategies, and predictive biomarkers and models. Finally, we consider clinical translation in cervical cancer populations, emphasizing RE incidence and pathology, validated risk-prediction tools, and key challenges in bridging mechanistic insights to bedside implementation.

## 2 Core mechanistic axes of radiation enteritis

### 2.1 DNA damage and repair dysfunction

Unrepaired DNA double-strand breaks (DSBs) are a primary instigator of radiation enteritis (RE) (Hu Z. et al., 2025). Pelvic irradiation generates abundant DSBs in intestinal epithelial and endothelial cells; if the DNA damage response (DDR) cannot efficiently resolve these lesions, checkpoint arrest and p53-mediated apoptosis deplete crypt stem cells, drive villus atrophy, and compromise the mucosal barrier (Potten et al., 1994a; Liu et al., 2016). Because the gut epithelium renews rapidly, even a small burden of persistent stem-cell DSBs can precipitate catastrophic mucosal breakdown (Leibowitz et al., 2021). In rodent abdominal irradiation (≈10–15 Gy), diffuse crypt death emerges within 1–2 days, and inadequate DSB repair predicts later fibrotic remodeling, underscoring the long-term consequences of early DDR failure (Liu et al., 2016).

**Figure 1:**
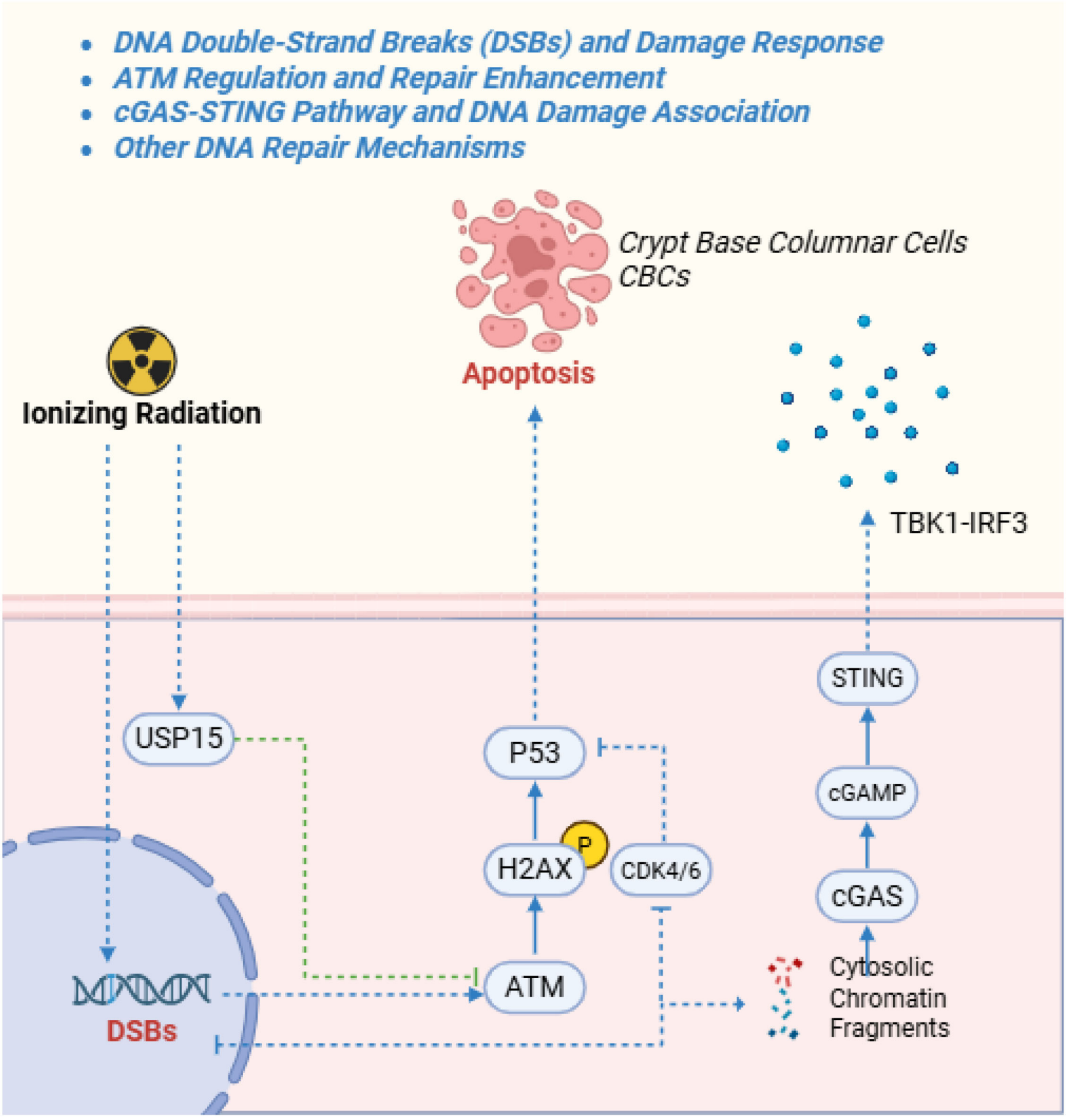
This schematic illustrates the core pathways of radiation-induced DNA double-strand breaks (DSBs) and the subsequent DNA damage response (DDR). Radiation leads to DSBs, activating ATM kinase, which phosphorylates H2AX and recruits repair proteins (e.g., Ku70/80, DNA-PKcs). Pathways branch into non-homologous end joining (NHEJ) or homologous recombination (HR) for repair, or p53-dependent apoptosis if unrepaired.

Ionizing radiation activates a DDR signaling network centered on ATM kinase. ATM phosphorylates histone H2AX (γ-H2AX) at break sites and propagates checkpoint signals through Chk2 and p53 to halt the cell cycle and coordinate repair-factor recruitment (Canman et al., 1998; Rajamanickam et al., 2017). In gut crypts, ATM activity is indispensable for genomic stability; loss of ATM function or insufficient activation—as may occur in older individuals or certain genetic backgrounds—exacerbates radiosensitivity, causing excess stem-cell apoptosis and failed mucosal regeneration (Ch’ang et al., 2005; Leibowitz et al., 2021) (**Figure 1**). Clinically, larger irradiated bowel volumes associate with higher rates of ≥Grade 2 toxicity, consistent with DDR capacity being overwhelmed across extensive intestinal segments (Kavanagh et al., 2010).

Multiple regulators fine-tune post-radiation repair. The deubiquitinase USP15, induced by irradiation, stabilizes ATM; USP15 knockout worsens intestinal inflammation and prolongs DSB persistence, whereas strengthening the ATM–USP15 axis preserves tissue architecture (Zhu et al., 2024b; Zhu et al., 2024a). Swertiamarin mitigates murine intestinal injury by lowering reactive oxygen species (ROS) and bile acid accumulation, indirectly reducing DNA damage signaling; by limiting bile-acid–driven hyperactivation of the cytosolic DNA sensor cGAS, it also restrains inflammatory amplification, highlighting DDR–redox–immune crosstalk (Xu et al., 2024; Zhao Z. et al., 2025).

Persistent DSBs initiate secondary injury programs. Cytosolic DNA fragments activate cGAS–STING, generating cGAMP and inducing type I interferons and NF-κB–dependent cytokines that recruit neutrophils and macrophages. Although protective during infection, sterile DNA sensing aggravates radiation mucosal damage and can drive chronic fibrosis if unchecked (Leibowitz et al., 2021; Xu et al., 2024). Endogenous restraints exist: T cells release extracellular ENPP1 to degrade cGAMP, and rectal delivery of ENPP1-enriched apoptotic vesicles accelerates healing by blunting interferon signaling downstream of cGAS (Zhou et al., 2024). In parallel, radiation can bias repair toward rapid but error-prone non-homologous end joining (NHEJ) over homologous recombination, potentially via PI3K signaling, leaving residual lesions and mutational scars (Ibrahim et al., 2012; Genetta et al., 2023). Transient pharmacologic G1 arrest with CDK4/6 inhibitors can improve crypt survival by slowing cycling and reducing conversion of p53 signaling into full apoptosis (Wei et al., 2016). Overall, RE initiation reflects DSB burden and DDR proficiency, while progression is shaped by apoptotic thresholds and containment of cGAS–STING–driven inflammatory spillover.

### 2.2 Oxidative stress and reactive oxygen species

**Figure 2:**
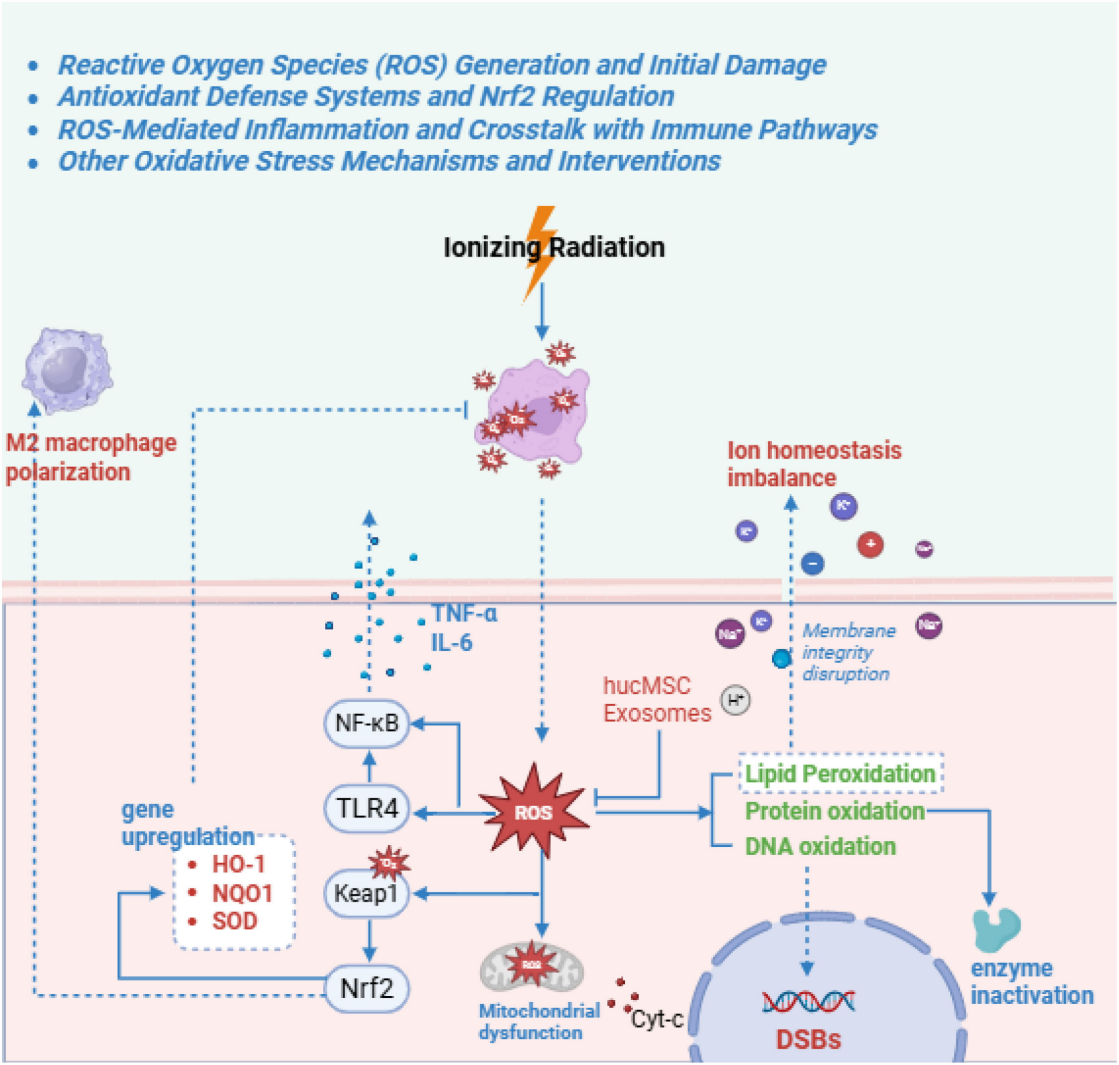
This diagram depicts radiation-induced reactive oxygen species (ROS) generation and downstream oxidative damage. Ionizing radiation ionizes water molecules, producing ROS, leading to lipid peroxidation, protein oxidation, and DNA damage. Antioxidant defenses are activated via Keap1-Nrf2 dissociation, resulting in nuclear translocation of Nrf2. Inflammation crosstalk occurs through TLR4 activation, amplifying cytokine release. Interventions enhance Nrf2 and inhibit ROS amplification loops.

Oxidative stress is a central driver of RE, especially early after irradiation (Shukla et al., 2016). Water radiolysis generates ROS (·OH, O_2_^−^, H_2_O_2_) that damage lipids, proteins, and DNA, causing lipid peroxidation, protein dysfunction, and additional strand breaks (Raghunath et al., 2018) (**Figure 2**). In the gut, this oxidative burst rapidly disrupts epithelial integrity, shortens villi, and increases permeability to luminal microbes and toxins (Shukla et al., 2016). Rodent studies show elevations of myeloperoxidase and nitrotyrosine, reflecting neutrophil infiltration and nitric oxide–derived oxidant production (Hussain et al., 1995). Clinically, high baseline oxidative stress or reduced antioxidant reserves—common in diabetes, metabolic syndrome, and advanced age—increase vulnerability, consistent with weakened glutathione and superoxide dismutase/catalase buffering (Sekhar et al., 2011a; Sekhar et al., 2011b).

The principal endogenous defense is the Nrf2 pathway. Under basal conditions, Nrf2 is sequestered by Keap1; ROS trigger Nrf2 release and nuclear translocation, inducing antioxidant and detoxification genes (e.g., HO-1, NQO1, and glutathione synthesis enzymes) (Itoh et al., 1997; Itoh et al., 1999). Intestinal Nrf2 is required for radioprotection: knockout mice develop profound villus atrophy, crypt loss, and heightened cytokine production after irradiation, whereas Nrf2 activation preserves mitochondrial function and limits epithelial and endothelial apoptosis (Jin et al., 2015). Nrf2 intersects with innate immunity; for example, the Nrf2 target Pirin suppresses cGAS expression, so Nrf2 deficiency can amplify cGAS–STING activity, inflammasome signaling, and pyroptotic cell death, reinforcing the ROS–inflammation feed-forward loop (Deniz et al., 2015; Zhou et al., 2024).

Enhancing Nrf2 signaling is therefore therapeutically attractive. Formononetin restores tight-junction proteins (Claudin-1, Occludin, ZO-1), reduces crypt apoptosis, and improves angiogenesis (increased CD31^+^ microvessels) after pelvic irradiation (Shukla et al., 2016; Hu Z. et al., 2025). α-Lipoic acid and calcitriol similarly activate Nrf2/HO-1 and glutathione programs to mitigate oxidative and inflammatory injury (Omer et al., 2022). With localized delivery or carefully timed dosing, transient Nrf2 activation in gut tissue need not protect tumors, supporting translational potential (Agrawal and Agrawal, 2019).

Exogenous antioxidants complement endogenous defenses. Ferulic acid activates the DJ-1/Nrf2/Sirt1 cascade, inhibits NF-κB and NLRP3 inflammasome activation, and reduces IL-1β and IL-18 release (Shukla et al., 2016; Zhang M. et al., 2025). EGCG neutralizes free radicals, stabilizes heat-shock proteins, and suppresses caspase-dependent apoptosis (Zhang et al., 2022). CAPE reduces p38 MAPK and ICAM-1 upregulation and lowers myeloperoxidase and nitric oxide levels, providing combined antioxidative and anti-inflammatory activity (Wang et al., 2025a). Nanomedicine aims to enhance mucosal specificity: amifostine loaded into chitosan nanoparticles concentrates thiol radioprotection in gut while limiting systemic toxicity, and Janus-type Ag/Ag_2_S nanostructures have been designed as tissue-selective ROS scavengers (Zhang M. et al., 2025).

Redox homeostasis is closely linked to the microbiome. Butyrate-producing Firmicutes support epithelial antioxidant capacity and immune balance via SCFAs, yet pelvic radiotherapy depletes these taxa and promotes blooms of facultative, pro-oxidant Proteobacteria (Sokol et al., 2008; Ma C-y. et al., 2025). Environmental stressors can exacerbate this loop; selected microplastics penetrate intestinal mucus, activate PI3K/Akt signaling to heighten epithelial radiosensitivity, and skew microbiota toward pro-oxidant profiles, increasing crypt loss (Xie et al., 2020). Thus, mitigating oxidative stress likely requires integrated strategies combining pharmacologic ROS control, diet-based redox support, and microbiota stabilization.

### 2.3 Immune-inflammatory cascades

**Figure 3:**
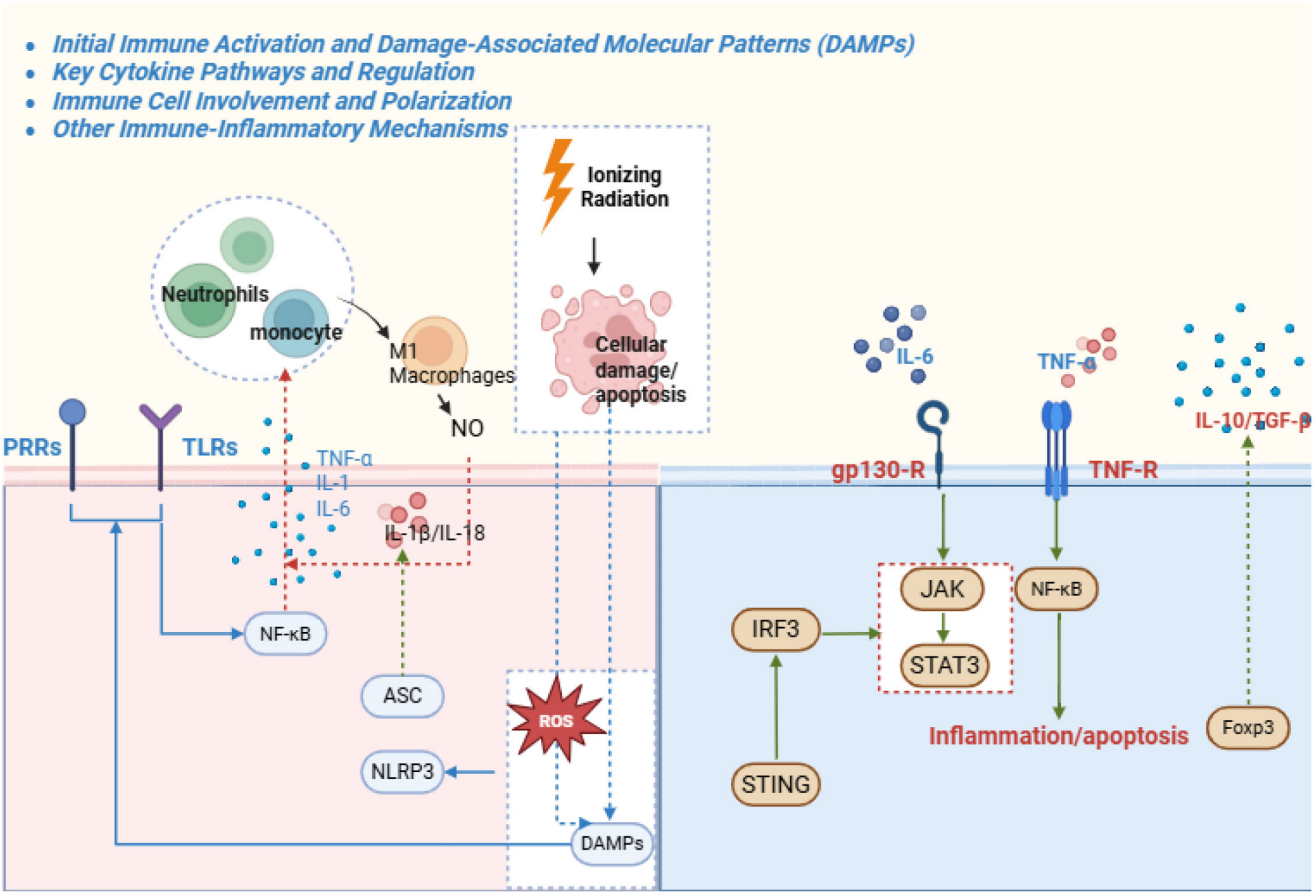
This network diagram outlines radiation-triggered immune activation via damage-associated molecular patterns (DAMPs) release, engaging TLRs and PRRs to activate NF-κB and JAK-STAT pathways. Key cytokines (TNF-α, IL-1β, IL-6) drive inflammation, with branches to immune cell recruitment (neutrophils, M1 macrophages) and polarization. Negative regulators include SOCS proteins and Nrf2-upregulated IL-10. Crosstalk with cGAS-STING (IFN production) and NLRP3 inflammasome (IL-1β/IL-18 maturation) is emphasized.

RE is an immune-driven inflammatory disease in addition to direct cytotoxicity (Wang Y-T. et al., 2025). Irradiated intestinal cells release damage-associated molecular patterns (DAMPs)—HMGB1, extracellular ATP, nuclear DNA, and histones—that activate pattern-recognition receptors on resident immune cells (I et al., 2025; Sato et al., 2025). TLRs on macrophages and dendritic cells detect HMGB1 and DNA to trigger MyD88/NF-κB signaling, while cytosolic RIG-I–like receptors sense RNA fragments from dying cells (Nabet et al., 2017). Together with cGAS–STING activation by leaked DNA, these pathways induce TNF-α, IL-1β, IL-6 and chemokines, recruiting leukocytes and amplifying tissue injury (Nabet et al., 2017) (**Figure 3**).

Clinical observations support dose-linked immune activation (Hovdenak et al., 2000; Huang et al., 2021). In cervical cancer pelvic radiotherapy, mean rectal doses above ~40 Gy correlate with elevated mucosal TLR4 expression and dense neutrophil infiltration in biopsies, aligning with acute cytokine surges and symptoms (diarrhea, cramping) (Hovdenak et al., 2000; Huang et al., 2021). A central acute effector is NLRP3 inflammasome activation in intestinal immune cells and likely severely injured epithelium. DAMPs and ROS activate NLRP3, driving caspase-1 maturation of IL-1β and IL-18 and cleavage of gasdermin D (GSDMD), leading to pyroptosis and further cytokine release, thereby accelerating crypt destruction and mucosal breakdown (Hu et al., 2020; Zhang S-H. et al., 2025).

Radiation also biases adaptive immunity toward pro-inflammatory phenotypes. It promotes a TH17-dominant response (IL-17A, IL-17F, IL-22) with reduced regulatory T cells (Geha et al., 2017; Wang Y-T. et al., 2025). IL-17 amplifies neutrophil recruitment, drives M1 macrophage polarization, and suppresses goblet-cell mucus production, weakening barrier integrity; IL-6 and IFN-γ further reinforce inflammation (Geha et al., 2017; Wang Y-T. et al., 2025). IL-22 is context dependent: while often epithelial-reparative, very high IL-22 exacerbates experimental radiation injury by increasing DNA damage, epithelial apoptosis, and mortality, and IL-22 blockade improves crypt survival and organoid growth (Hamade et al., 2022; Wang J. et al., 2025). High baseline IL-22 has therefore been proposed as a biomarker of radiosensitivity and a cue for intensified monitoring or prophylactic anti-inflammatory strategies (Hamade et al., 2022; Wang J. et al., 2025).

Chronic RE reflects incomplete inflammatory resolution and progressive fibroinflammatory remodeling. Radiation induces senescence in fibroblasts, endothelial cells, and other stromal populations, and senescent cells sustain a SASP that maintains low-grade NF-κB and inflammasome signaling (Takeuchi et al., 2012; Wang Y-T. et al., 2025). Chronic lesions typically show persistent lymphocyte and macrophage infiltration that orchestrates fibrogenesis: macrophage-derived TGF-β activates fibroblasts, CD4^+^ cytokines maintain myofibroblast differentiation, and endothelial-to-mesenchymal transition (EndoMT) contributes to capillary loss and fibrosis (Takeuchi et al., 2012; Wang Y-T. et al., 2025). Proteomic studies of late lesions have reported upregulation of mediators such as CD13, EN-RAGE, and CCL28, consistent with ongoing immune recruitment (Ren et al., 2025).

Inflammatory architecture varies by histologic subtype. Telangiectatic radiation proctitis may feature granulomas and persistent macrophage infiltration, whereas ulcerative disease can show severe transmural inflammation with fistula formation reported in up to ~75% of deep ulcerations (Yu et al., 2023; Wang Y-T. et al., 2025). Macrophages may later adopt M2-like wound-healing phenotypes that secrete VEGF and fibroblast growth factors; these responses support repair and angiogenesis yet may also promote aberrant angiogenesis or fibrosis, and late-phase macrophage depletion impairs ulcer healing, illustrating the need to preserve reparative immunity while restraining destructive inflammation (Yu et al., 2023; Wang Y-T. et al., 2025).

Therapeutic implications favor calibrated immunomodulation. Candidate suppressors include TLR4/MyD88 inhibitors, STING antagonists, and NLRP3 or caspase-1 blockade (Sánchez-Aragó et al., 2013). Pro-repair strategies include MSCs, which secrete IL-10, PGE_2_, and TGF-β3 to shift TH17/M1 toward Treg/M2; adipose-derived MSCs reduce IL-17A, expand FoxP3^+^ Tregs, and improve histology in rodents (Németh et al., 2009). Probiotics can modulate mucosal immunity; Lactobacillus rhamnosus GG inhibits STING signaling and limits inflammatory infiltration (Zhang L-L. et al., 2024). For chronic disease models, Compound Kushen injection attenuates fibrosis via cannabinoid receptor 1–mediated suppression of NOX4/NF-κB and CD68^+^ macrophages, while osteopontin (OPN) loss reduces chronic inflammation and fibrosis but can impair angiogenesis needed for healing, emphasizing trade-offs in target selection (Zhang L-L. et al., 2024; Si et al., 2025; Xu et al., 2025).

### 2.4 Epithelial barrier damage and stem cell loss

The intestinal epithelium is a single-cell barrier reinforced by tight junctions and continuously replenished by intestinal stem cells (ISCs) (Furuse et al., 1998). Radiation disrupts this system early and profoundly (Shukla et al., 2016). Even moderate doses (~10–12 Gy in animal models) induce villus blunting, crypt shrinkage, and near-complete suppression of proliferation within 1–3 days (Shukla et al., 2016). In fractionated pelvic radiotherapy, barrier leakiness enables endotoxin and bacterial translocation; elevated serum endotoxin and permeability assays (e.g., fluorescein or FITC-dextran) correlate with inflammatory markers such as myeloperoxidase and IL-6, and complications including ileus or bacteremia can occur in severe cases (Shukla et al., 2016; Banerjee et al., 2019).

Tight-junction (TJ) disassembly is a defining lesion (Shukla et al., 2016). Claudins, occludin, and ZO-1 are internalized or degraded, creating paracellular gaps; Claudin-3 is particularly important, and its loss predicts bacterial translocation and systemic infection risk. Barrier-repair strategies show benefit in models: neurotensin upregulates Claudin-3 and helps reseal TJs, whereas rebamipide stabilizes β-catenin and claudin proteins to promote epithelial regeneration and reduce cytokine release (Joshi et al., 2014; Shukla et al., 2016).

Microvascular injury further constrains repair. Endothelial apoptosis and capillary dropout cause mucosal ischemia, contributing to chronic RE, including fragile telangiectasias prone to bleeding (Han et al., 2017; Toullec et al., 2018). Endothelial-protective agents are promising: Ecliptasaponin II activates PPARγ to prevent endothelial mitochondrial oxidative injury (an effect lost with PPARγ blockade), and geranylgeranylacetone induces endothelial HSP70 and nitric oxide signaling to mitigate radiation-induced apoptosis (Han et al., 2017; Zeng et al., 2025).

**Figure 4:**
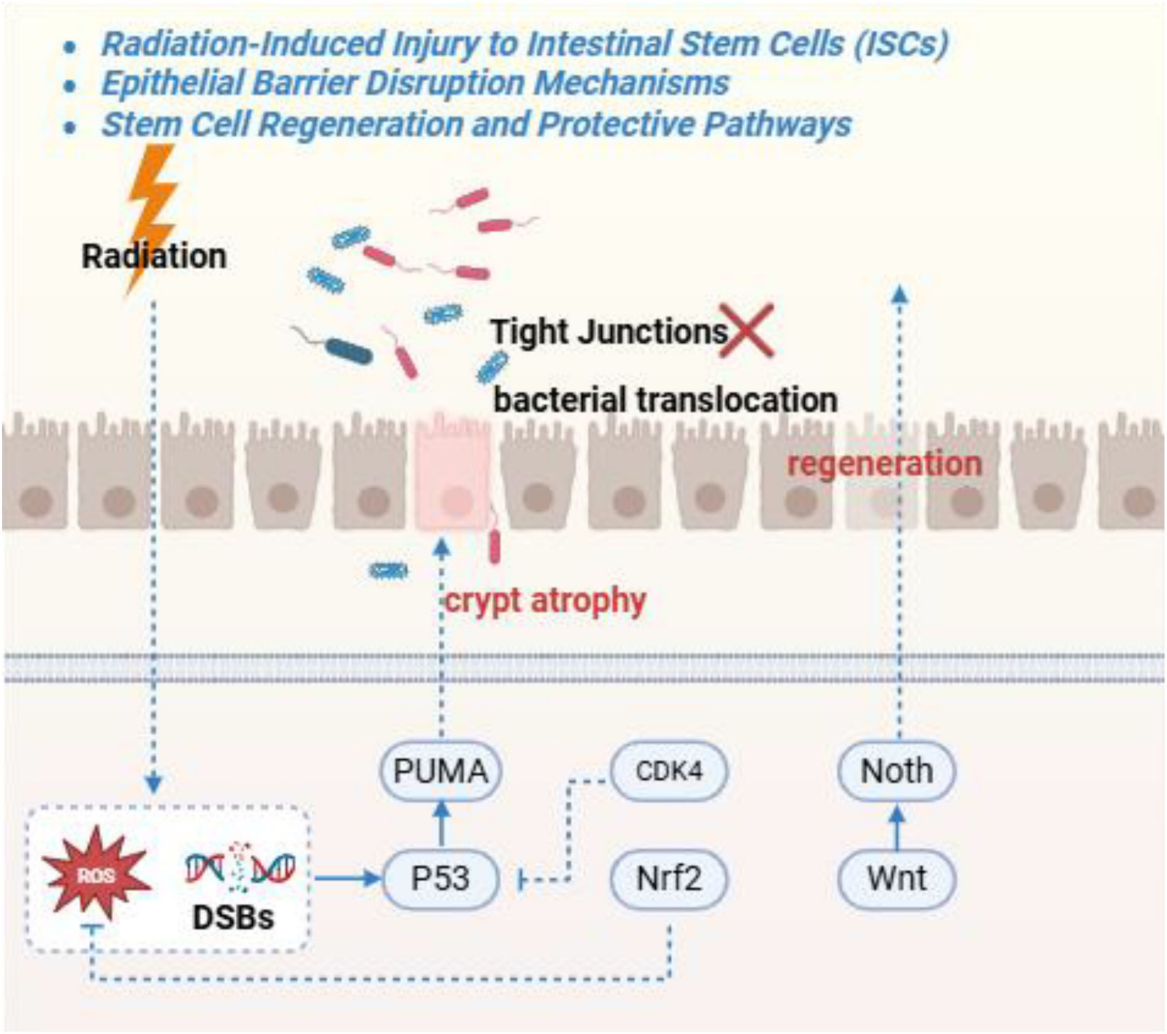
This Figure shows radiation-induced injury to intestinal stem cells via DSBs/ROS, activating p53-PUMA for apoptosis and Wnt inhibition for proliferation arrest. Downstream effects include tight junction disruption and mucus layer thinning. Regeneration pathways involve Wnt, Notc. Inflammation exacerbating barrier permeability and bacterial translocation.

ISC preservation is central to recovery. Lgr5^+^ crypt base ISCs are highly radiosensitive due to rapid cycling and Wnt dependence; >80% can undergo apoptosis within 24 hours after moderate exposure, producing acute mucosal atrophy (Zitter et al., 2023) (**Figure 4**). When ISC depletion exceeds regenerative capacity, re-epithelialization fails (Chivukula et al., 2014). Radiation perturbs ISC regulation by suppressing Wnt/β-catenin (via p53-induced Wnt antagonists) and hyperactivating Notch (Liu et al., 2016). Dying cells release signals, including small RNAs, that activate Notch likely through RIG-I, pushing progenitors toward an undifferentiated state and accelerating loss of Lgr5^+^ cells; RIG-I deficiency blunts Notch hyperactivation and improves crypt regeneration, suggesting RIG-I inhibition or γ-secretase blockade as candidate protective strategies (Bohin et al., 2020; Zhao X. et al., 2025).

Intrinsic regulators further shape radioresilience. BVES restrains Wnt and Notch intensity; Bves knockout accelerates crypt regrowth in a Wnt ligand–dependent manner (Reddy et al., 2016). MTG16 promotes p53-mediated ISC apoptosis; MTG16 deletion accelerates DNA repair and improves crypt survival (Short et al., 2024). KLF5 influences repair competence: Klf5 deficiency impairs DNA repair and increases ISC loss, whereas controlled KLF5 overexpression enhances regeneration without increasing tumor risk, suggesting genotype-dependent radiosensitivity (Li et al., 2015; Wang et al., 2025b).

Therapeutically, strategies aim to protect ISCs during radiotherapy and accelerate regeneration afterward. R-spondin1 (RSPO1), a potent Wnt agonist, expands Lgr5^+^ cells; systemic or MSC-mediated RSPO1 delivery accelerates regeneration in irradiated mice (Bhanja et al., 2009). Fecal virome transplantation (FVT) enhances ISC recovery by suppressing RIG-I/Notch overactivation, increasing organoid growth, and reducing crypt apoptosis (Zhao X. et al., 2025). HSP90 inhibitors (e.g., AUY922) may protect intestinal progenitors while shifting the microbiota toward SCFA-producing taxa, reducing inflammation and improving crypt survival (Yoshimura et al., 2025). MSC infusion also improves injury in large-animal models and increases anti-inflammatory and trophic mediators (e.g., IL-10, KGF, FGF), supporting epithelial repair (François et al., 2012).

### 2.5 Programmed cell death: apoptosis, ferroptosis, and pyroptosis

Radiation-induced intestinal injury engages multiple programmed cell-death (PCD) pathways. Apoptosis, ferroptosis, and pyroptosis contribute in distinct temporal and cellular contexts, frequently co-activating to drive acute crypt loss and chronic remodeling (Thermozier et al., 2020; Ji et al., 2022).

Apoptosis dominates the acute phase. DNA damage activates p53 in crypt progenitors and ISCs, inducing PUMA, Bax, and Bak and repressing Bcl-2, leading to mitochondrial outer-membrane permeabilization, cytochrome c release, and caspase-9→caspase-3 activation (Potten et al., 1994b; Liu et al., 2016). Within 6–24 hours after moderate exposure (~12 Gy in rodents), up to ~80% of crypt cells undergo apoptosis (Potten et al., 1994b). Endothelial apoptosis contributes to microvascular failure; reduced Hey2 limits endothelial apoptosis and EndoMT, preserving perfusion (Erdreich-Epstein et al., 2005). Because baseline apoptosis removes irreparably damaged cells, inhibition must be selective and time-limited (Scott, 2014). PPARγ agonists (rosiglitazone or pioglitazone) blunt mitochondrial apoptosis by lowering the Bax/Bcl-2 ratio and cytochrome c release; transient CDK4/6 arrest is similarly crypt-sparing (Hu et al., 2020). Broad caspase inhibitors (e.g., z-VAD-fmk) or IAP mimetics show crypt protection in models but require caution to avoid preserving genomically unstable cells (Loriot et al., 2014).

Ferroptosis is an iron-dependent PCD defined by lethal lipid peroxidation and is increasingly implicated in RE, particularly in the endothelium and in chronic lesions (Xie et al., 2022). Radiation increases ROS, depletes glutathione, impairs GPX4, and induces ACSL4, sensitizing polyunsaturated membrane lipids to peroxidation; 4-HNE adducts and excess free iron accumulate in irradiated intestine (Zhang L-M. et al., 2024; Cao et al., 2025). Endothelial ferroptosis drives capillary dropout and chronic ischemia, and iron deposition with lipid peroxidation products in fibrotic lesions suggests links to fibroblast activation and senescence (Xie et al., 2022). Anti-ferroptotic strategies include pioglitazone, which diverts glucose into the hexosamine pathway to stabilize ferritin heavy chain and reduce transferrin receptor expression, shrinking the labile iron pool; selenium nanoparticle–engineered E. coli Nissle can act as an oral GPX4 mimic; and coniferyl aldehyde suppresses ACSL4 while inducing HSP70, limiting membrane peroxidation (Liu et al., 2020; Thermozier et al., 2020).

Pyroptosis is an inflammatory, lytic PCD initiated by inflammasomes and prominent in infiltrating macrophages and severely injured epithelium (Gu et al., 2022). Radiation-induced DAMPs and ROS activate NLRP3, leading to caspase-1 cleavage of pro-IL-1β/pro-IL-18 and GSDMD; GSDMD pores drive swelling and rupture, releasing IL-1β and IL-18 that recruit neutrophils and amplify mucosal injury (Fenini et al., 2020; Okano et al., 2024). Multiple agents restrain this axis: corilagin restores glutathione and blocks NLRP3–caspase-1–GSDMD, cynaroside inhibits dynamin-2 to prevent inflammasome assembly, and ferulic acid further restrains pyroptosis by activating Sirt1 and inhibiting NF-κB, destabilizing inflammasome adaptor specks and reducing gasdermin pore formation (Hu et al., 2020; Zhang S-H. et al., 2025).

These PCD modes are coupled: apoptotic debris supplies DAMPs that activate inflammasomes, ferroptotic oxidized lipids can recruit and activate immune cells, and endothelial death worsens hypoxia, provoking secondary epithelial apoptosis (Hong et al., 2017). Consequently, indiscriminate blockade of a single pathway may be counterproductive; the goal is balanced, context-dependent modulation that limits excessive crypt apoptosis, protects vasculature from ferroptosis, and tempers inflammasome-driven pyroptosis without compromising tumor radiosensitivity.

### 2.6 Microbiota dysbiosis and metabolite signaling

Gut microbiota dysbiosis is now recognized as a mechanistic contributor to RE rather than a downstream epiphenomenon (Wang et al., 2025c). Pelvic radiation rapidly reduces microbial diversity and shifts fecal composition, consistently depleting SCFA-producing Firmicutes, especially butyrate-producing genera (Patel et al., 2025). Butyrate fuels colonocytes and supports anti-inflammatory programs (including Treg differentiation and barrier reinforcement); its loss lowers SCFA availability, weakens epithelial integrity, and biases mucosal immunity toward inflammation (Machiels et al., 2016; Ferrer-Picón et al., 2020). Radiation can also promote expansion of opportunistic Enterobacteriaceae/Enterococcus and increase luminal phenylethylamine, while perturbing tryptophan metabolism; an “indole-3-ethanol imbalance” has been described, and indole-3-ethanol supplementation alleviates RE in mice, implicating loss of specific metabolites as functional drivers (Xiao et al., 2020; Cao et al., 2022).

These microbial shifts feed forward into injury. Dysbiosis can elevate oxidative stress via microbes that generate hydrogen peroxide or nitrosamines, raising mucosal ROS (El-Shenawy et al., 2017). Barrier disruption enables LPS translocation, activating TLR4 on lamina propria macrophages and sustaining NF-κB–dependent cytokine release (Lotz et al., 2007). Loss of SCFA-producing communities also removes proliferative cues for ISCs, slowing epithelial regeneration (Farhadipour et al., 2023). A 2025 cervical-cancer multi-omics study reported that baseline microbiome features predict acute RE severity: enrichment of Faecalibacterium and other SCFA producers associates with resilience, whereas a pro-inflammatory baseline community increases risk; transfer studies further support a causal component (Ma C-y. et al., 2025).

Recent work has mapped specific metabolite–receptor–cytokine circuits. A 2024 study identified 3-hydroxybutyrate (3HB) as a protective microbial signal in radiation proctitis: fecal and serum 3HB fall after irradiation and inversely correlate with mucosal IL-6 and injury; exogenous 3HB suppresses GPR43 (FFA2)–mediated IL-6 signaling and improves histopathology (Ge et al., 2024). Akkermansia muciniphila is a dominant 3HB source; irradiation reduces Akkermansia in mice and patients, and oral Akkermansia restores 3HB, dampens GPR43/IL-6 activation, and mitigates mucosal injury (Ge et al., 2024). Another 2024 study identified indole-3-carboxaldehyde (I3A), a Lactobacillus-derived tryptophan metabolite, as a radiomitigator: I3A activates AhR in epithelial and immune cells, induces IL-10 and Wnt signaling, enhances Lgr5^+^ ISC proliferation, improves barrier integrity, and does not protect colorectal tumor cells, supporting therapeutic selectivity (Xie et al., 2024).

Diet can be leveraged to engineer protective microbiota–metabolite outputs. A 2025 study showed that phytate, abundant in whole grains and legumes and poorly absorbed by humans, promotes Parasutterella colonization in mice (Li et al., 2025). Parasutterella produces 3-phenyllactic acid (PLA) and N-acetyl-L-leucine (NL): PLA promotes anti-inflammatory macrophage polarization (M2-like), whereas NL reduces ROS and lipid peroxidation; together they improve GI integrity and hematopoietic recovery, although high-dose phytate may impair mineral absorption, necessitating dosing safeguards (Shakya et al., 2022; Li et al., 2025).

Broader restoration strategies are also under study. Fecal microbiota transplantation (FMT) can replenish SCFA-producing taxa, raise luminal SCFAs, and reduce inflammatory markers; reported benefits include improved crypt survival with suppression of aberrant RIG-I/Notch signaling and restoration of indole pathways (Xiao et al., 2020; Zhang et al., 2023). Fecal virome transplantation (FVT) similarly promotes ISC regeneration by dampening RIG-I hyperactivation (Zhao X. et al., 2025). However, efficacy is strain- and context-dependent: a randomized trial of Saccharomyces boulardii improved stool consistency but did not significantly reduce overall RE incidence or severity, underscoring that probiotics are not interchangeable and must be mechanism matched (Eren et al., 2010).

In sum, dysbiosis both marks and drives RE: loss of SCFA producers and protective metabolites weakens barrier and repair programs, whereas pro-inflammatory shifts amplify oxidative and immune injury. Mechanism-guided interventions—selective probiotics, prebiotics, postbiotic metabolites, FMT/FVT, and targeting pathways such as GPR43–IL-6 and AhR–IL-10/Wnt—offer opportunities for personalized prevention and mitigation.

## 3 Modulators and interventions targeting key mechanisms

### 3.1 Small molecules and natural products

#### 3.1.1 Antioxidants and redox modulators

Given the central role of ROS in RE, numerous antioxidant compounds have been examined. α-Lipoic acid (ALA), a mitochondrial antioxidant, attenuated acute RE in mice by inhibiting NF-κB and MAPK activation, reducing inflammatory-cell infiltration, and accelerating mucosal repair (Jeong et al., 2016; Kim et al., 2017). A pilot clinical study suggested that oral ALA may alleviate acute radiation enteropathy in prostate cancer patients, though larger trials are needed for confirmation (Farhood et al., 2020). Ferulic acid and caffeic acid phenethyl ester (CAPE), two polyphenols with antioxidant and anti-inflammatory properties, significantly lowered mucosal TNF-α, IL-1β, and oxidative-injury markers in rodent RE models (Jin et al., 2015). Ferulic acid also inhibits inflammasome activation and pyroptosis, supporting multi-target potential (Liu et al., 2022). EGCG (epigallocatechin-3-gallate, from green tea) protected intestinal crypts by suppressing caspase-3 activation and enhancing cytoprotective heat-shock responses (Xie et al., 2020). A small human study reported reduced rectal bleeding in chronic radiation proctitis after EGCG administration, although definitive clinical evidence remains limited (Tsai et al., 2017).

#### 3.1.2 Nrf2 activators

Targeting the Nrf2 antioxidant-defense axis has yielded candidate radioprotectors. Formononetin (an isoflavone from red clover) and swertiamarin (an iridoid from *Swertia* herbs) are natural Nrf2 activators that protected irradiated intestines in mice (Tang R. et al., 2025). Formononetin increased HO-1 and tight-junction protein expression, strengthening the epithelial barrier while limiting oxidative injury (Tang R. et al., 2025). Dimethyl fumarate (DMF), an FDA-approved Nrf2 activator for other indications, represents another option, although its gastrointestinal side effects mean localized or low-dose delivery would be preferable in RE (Yoshimura et al., 2025). Sulforaphane, a broccoli-derived Nrf2 inducer, shows general radioprotective activity *in vitro* but has not yet been tested in RE models (Wei et al., 2021). Because sustained systemic Nrf2 activation could theoretically protect tumors or disrupt normal homeostasis, emphasis is on transient, tissue-specific delivery—for example, orally or rectally targeted formulations that activate Nrf2 in gut mucosa during radiotherapy without substantial systemic exposure (Occhiuto et al., 2023).

#### 3.1.3 Anti-inflammatory and immune modulators

Inflammation drives both acute injury and chronic fibrotic evolution, so numerous anti-inflammatories have been evaluated. 5-Aminosalicylic acid (5-ASA) showed modest benefit in a rat model of radiation proctitis and greater efficacy when combined with probiotic LGG (Dandin et al., 2015). Clinically, prophylactic 5-ASA has shown inconsistent benefit in pelvic radiotherapy; systematic review evidence found limited effect when 5-ASA was used alone (Lawrie et al., 2018). Corticosteroids can control severe acute enteritis, but systemic side effects (immunosuppression, metabolic complications) limit prophylactic use (Feuerstein et al., 2025). Statins, owing to anti-inflammatory and endothelial-protective actions, have shown promise experimentally; pravastatin reduced acute radiation enteropathy and improved tight-junction integrity in mice (Jang et al., 2018). More targeted biologics and pathway-specific inhibitors are being explored: TNF-α blockers (e.g., infliximab) could theoretically reduce severe proctitis but raise concerns regarding infection risk and impaired healing (Pineton de Chambrun et al., 2020). Preclinical interleukin antagonism suggests that blocking IL-1 or IL-6 may reduce the acute cytokine surge associated with symptoms (Yang Y. et al., 2023). As noted, an IL-22 binding protein improved outcomes in a mouse model, consistent with evidence that excessive IL-22 can exacerbate injury (Hanash et al., 2012). Another approach is inhibiting MyD88, an adaptor for TLR signaling, to prevent radiation-induced innate immune activation, though no MyD88 inhibitors have entered clinical trials for RE (Medler et al., 2023).

#### 3.1.4 Cell-death modulators

Several small molecules aim to modulate programmed-cell-death pathways central to RE. PPARγ agonists (rosiglitazone, pioglitazone) have anti-apoptotic and anti-ferroptotic effects in gut tissue; pioglitazone reprogrammed cellular metabolism and protected crypts from ferroptosis in mice, though systemic metabolic effects (e.g., insulin sensitivity changes) favor localized delivery (e.g., enema formulations) (Hu et al., 2020; Zeng et al., 2025). Direct ferroptosis inhibitors such as liproxstatin-1 have shown efficacy mainly in cell culture models (Friedmann Angeli et al., 2014). Caspase inhibitors (e.g., the pan-caspase inhibitor z-VAD-fmk) can suppress apoptosis (caspase-3–mediated) and pyroptosis (caspase-1–mediated), enhancing crypt survival in rodents, but systemic inhibition risks disturbing turnover and immune function (Wei et al., 2016). Dynasore, a dynamin inhibitor, blocked NLRP3 inflammasome assembly in a mouse model and reduced intestinal pyroptosis (Shan et al., 2024). VX-765, a selective caspase-1 inhibitor tested in humans for other indications, is a candidate for repurposing to target inflammasome-driven injury in RE (Wannamaker et al., 2007).

#### 3.1.5 Nanoparticle drug-delivery systems

Advanced delivery platforms can improve efficacy while reducing systemic exposure. A 2025 study developed a nanoprobe that senses intestinal ROS surges (near-infrared II fluorescent signaling) and releases antioxidants in real time (Zhang M. et al., 2025). Another group engineered amifostine-grafted chitosan nanoparticles to selectively deliver amifostine to gut tissues; this enhanced mucosal protection while minimizing dose-limiting systemic side effects such as hypotension (Qian et al., 2025). These examples illustrate how “smart,” cue-responsive delivery triggered by pathological ROS can support precision mitigation of RE without additional systemic toxicity.

#### 3.1.6 Herbal and traditional medicine extracts

Multi-component herbal formulations used in Chinese medicine have shown mechanistic benefits in experimental RE. Huanglian Decoction alleviated RE by modulating ER stress and inflammasome pathways (Shi et al., 2025). Liangxue Guyuan Yishen Decoction (LGYD) improved survival after lethal abdominal irradiation by activating Hes1/STAT3 signaling and expanding the intestinal stem-cell (ISC) pool, suggesting pro-regenerative effects on crypt cells (Yan et al., 2023). Compound Kushen Injection reduced chronic radiation inflammation and fibrosis via CB1-mediated pathways (Xu et al., 2025). Researchers have also isolated bioactive constituents: Ecliptasaponin II (from *Eclipta*) protects endothelial cells via PPARγ activation; bixin (from annatto) has potential anti-fibrotic activity; emodin (from rhubarb and related plants) has been associated with barrier protection and reduced apoptosis (Wang et al., 2016; Xue et al., 2018; Zeng et al., 2025). Their pleiotropic actions may be advantageous in RE, where multiple mechanisms intersect.

#### 3.1.7 Evidence and development stage

Most small-molecule and natural-product interventions remain at cell-culture or rodent stages of evidence. Limited human data exist for amifostine (historically used as a radioprotector), statins, and probiotic plus 5-ASA combinations, but none are routine clinical practice for RE (Athanassiou et al., 2003; Dandin et al., 2015). Translation from animal models to fractionated human radiotherapy requires caution given differences in schedules, drug metabolism, and host–microbiome interactions. Nonetheless, convergence on key pathways supports these targets, with the central challenge being safety and feasibility in cancer patients without compromising tumor control.

### 3.2 Cell-based therapies and bioengineered solutions

#### 3.2.1 Mesenchymal stromal cells

MSC-based therapy is an advancing strategy for radiation-induced normal-tissue injury because of immunomodulatory and regenerative activity. MSCs derived from adipose tissue, bone marrow, umbilical cord, or amniotic membrane consistently benefit models of radiation enteropathy. After systemic administration, MSCs home to injured gut sites via chemokine cues and secrete anti-inflammatory cytokines (e.g., IL-10, TGF-β3), trophic factors (e.g., keratinocyte growth factor, VEGF, R-spondin1), and matrix-remodeling enzymes. In acute RE models, adipose-derived MSCs reduced mucosal TNF-α and IL-17, increased regulatory T-cell populations, and enhanced crypt survival. In chronic/fibrosis models, MSC therapy attenuated collagen deposition and preserved villus structure, partly by suppressing EndoMT and vascular inflammation, reflected by reduced ICAM-1/VCAM-1 expression in intestinal vessels.

Large-animal studies lend translational support. In beagle dogs receiving fractionated abdominal irradiation, angiographically delivered autologous MSCs roughly halved the histopathologic injury score and increased local IL-10, demonstrating feasibility in an anatomically relevant setting. MSCs can be used autologously or allogeneically given low immunogenicity; allogeneic bone-marrow MSCs are already used clinically for steroid-refractory graft-versus-host disease. Early clinical experience and Phase I/II trials of MSC infusions for chronic radiation proctitis suggest local (endoscopic or intra-arterial) and systemic administration are generally well tolerated, with preliminary symptom signals (e.g., reduced rectal bleeding or diarrhea). Delivery optimization remains important: IV infusion may lead to pulmonary trapping, whereas mesenteric arterial injection or endoscopic submucosal injection may improve homing.

#### 3.2.2 MSC-derived vesicles

Increasing attention focuses on MSC-derived vesicles as cell-free alternatives. Exosomes and apoptotic bodies carry miRNAs, proteins, and enzymes that reproduce many MSC effects without risks of live-cell transplantation. Preclinical studies show MSC extracellular vesicles can attenuate radiation-induced intestinal injury and enhance epithelial recovery, limiting oxidative stress and inflammation while improving DNA repair responses (reduced γ-H2AX foci). ENPP1 enrichment within apoptotic bodies promoted cGAMP degradation, mitigating STING-mediated inflammatory signaling. MSC exosomes also activate the Nrf2/HO-1 pathway in target cells. These vesicles could potentially be produced as off-the-shelf biologics and delivered locally (enema/suppository), simplifying administration.

Beyond cell transplantation, growth factors offer complementary strategies. R-spondin1 expands ISCs via Wnt signaling. IL-22 and the IL-22/IL-22BP axis have been investigated, but therapeutic use would require maintaining IL-22 within a beneficial range. Palifermin (keratinocyte growth factor), FDA-approved to prevent oral mucositis, reduced ulceration and increased crypt depth in a rat radiation proctitis model. In Japan, basic fibroblast growth factor (FGF2) has been applied locally (enema or submucosal injection) for chronic radiation proctitis with encouraging mucosal-healing results.

Given the role of microvascular injury in chronic RE, endothelial progenitor cells (EPCs) and pro-angiogenic approaches are under study. Bone-marrow EPC transplantation improved angiogenesis and reduced fibrosis in animal models. Local VEGF delivery or gene therapy to augment angiogenic factors can attenuate late injury experimentally, but pro-angiogenic strategies must be balanced against theoretical risks of stimulating residual tumor vasculature; localized application is therefore emphasized.

Bioengineered Tissue Constructs: Regenerative medicine approaches such as tissue engineering are being considered. Transplantation of laboratory-grown intestinal organoids/enteroids has engrafted functional intestinal epithelium in mouse colon, suggesting that autologous ISC expansion and endoscopic/surgical delivery could repair focal ulcers or strictures, although this remains early-stage. Other innovative directions include microbiome-targeted viral therapies, such as commensal bacteriophages to eliminate radiation-amplified pathobionts (e.g., *Enterococcus faecalis*) or deliver beneficial genes (e.g., ROS-detoxifying enzymes) to reshape the microbial ecosystem.

Overall, cell-based and bioengineered therapies are less clinically mature than small molecules. Evidence is predominantly preclinical or veterinary with limited early human signals. Priorities include scalable manufacturing, rigorous safety assessment (especially for live or gene-modified products), and controlled trials demonstrating durable benefit.

### 3.3 Gut microbiota and microbial metabolite therapies

#### 3.3.1 Conventional probiotics

A variety of probiotic strains have been tested. LGG is among the best studied; prophylactic LGG reduced histologic damage, preserved crypt–villus morphology, and dampened inflammation in mice (Zhang L-L. et al., 2024). Mechanistically, LGG exopolysaccharides can inhibit STING signaling and reduce M1 macrophage polarization (Si et al., 2025). In rats, LGG combined with 5-ASA improved healing of radiation proctitis lesions relative to either agent alone (Dandin et al., 2015). Clinically, small trials testing probiotics during pelvic radiotherapy have been mixed: some multi-strain mixtures (often *Lactobacillus*/*Bifidobacterium*) reduced diarrhea and bloating, whereas others reported no significant benefit (Delia et al., 2007). Meta-analyses up to 2020 tentatively suggested probiotics may reduce ≥Grade 2 acute diarrhea, but heterogeneity in strains, dosing, endpoints, and concurrent therapies limits firm conclusions (Liu et al., 2017). Probiotics for established chronic RE have generally been disappointing, consistent with fibrosis and vascular damage being less reversible by microbiome modulation alone (Jo et al., 2024).

Strain specificity is emphasized by divergent results. A spore-forming *Bacillus coagulans* strain (BC99) showed strong effects in a 2025 mouse study: oral BC99 restored Lachnospiraceae abundance and SCFA production, reduced crypt dropout and pro-inflammatory cytokines, and improved survival after abdominal irradiation; spore formation may enable more reliable delivery and persistence (Tang LF. et al., 2025). In contrast, *Saccharomyces boulardii* did not significantly prevent acute radiation enteritis in a randomized trial (Muangwong et al., 2025). Disparate outcomes likely reflect differences in strain function, dosing and duration, baseline microbiome composition, antibiotic exposure, and diet.

#### 3.3.2 Engineered probiotics

Engineered commensals extend beyond natural strain capabilities. “AAEcN,” an *E. coli* Nissle 1917 derivative engineered to overproduce superoxide dismutase and catalase, lowered intestinal ROS, improved crypt survival, and enhanced bone-marrow recovery after irradiation in mice (Wang et al., 2025d). Adhesive curli fibers fused to a TFF3-derived gut-homing peptide promoted mucosal adherence, forming a temporary “living bandage.” (Wang et al., 2025d) Another design, EcN-Se@SA, loads *E. coli* Nissle with selenium nanoparticles and uses an acid-resistant coat; selenium acts as a GPX4 mimic to curb ferroptosis while modulating immunity and microbiota balance (Sun et al., 2025). Safety concerns motivate kill-switch circuits and antibiotic sensitivity to enable eradication.

#### 3.3.3 Microbial metabolite supplementation (postbiotics)

Postbiotic supplementation targets metabolite mediators of microbiome benefit. Butyrate enemas have been used in chronic radiation proctitis with reports of reduced bleeding and pain, plausibly via colonocyte fueling, barrier reinforcement, and repair promotion, though adherence can be challenging (Stojcev et al., 2013; Maggio et al., 2014). Additional candidates include 3-hydroxybutyrate (3HB) for IL-6 suppression and GPR43-mediated radioprotection, indole-3-carboxaldehyde (I3A) to activate AhR and boost IL-10/Wnt signaling, and PLA/NL metabolites associated with phytate’s effects (Ge et al., 2024). Practical barriers include pleiotropy, short half-lives, and digestive modification; colon-targeted delivery (pH-dependent capsules, polymer coatings) may improve feasibility.

#### 3.3.4 Community replacement (microbiota transplant)

Evidence for fecal microbiota transplantation (FMT) in RE is limited to case reports and small series. Serial FMTs improved symptoms and partially healed mucosa in severe chronic radiation colitis; small proctitis series reported decreased bleeding in some patients with variable outcomes (Ding et al., 2020; Wang et al., 2024). A mechanistically appealing extension is sequencing-guided identification of beneficial engrafting strains and development of defined consortia (10–20 cultured commensals). Fecal virome transplantation (FVT) remains preclinical; a mouse proof-of-concept suggests phage components may aid stem-cell recovery after radiation (Zhao X. et al., 2025).

#### 3.3.5 Dietary strategies (prebiotics and functional foods)

Diet can modulate microbiota during radiotherapy. Fiber supports SCFA production but may worsen gas and bloating, so patients often reduce intake during acute symptoms (Then et al., 2024). Targeted fermentable fibers (resistant starch, selected β-glucans) may expand butyrate producers and be better tolerated, but personalization is needed (Jayachandran et al., 2018). Phytate may enrich Parasutterella and downstream PLA/NL production, yet high-dose phytate can impair mineral absorption (Li et al., 2025). Glutamine supplementation shows inconsistent trial results, likely reflecting dosing and baseline nutritional variability (Cao et al., 2017). Arginine remains controversial given potential systemic metabolic effects and theoretical tumor-support concerns (Lind, 2004).

#### 3.3.6 Evidence and translational readiness

Microbiome interventions span moderate clinical experience to early preclinical concepts. Conventional probiotics show modest preventive signals for acute RE (notably diarrhea reduction), but heterogeneity and strain dependence limit endorsement (Delia et al., 2007). Engineered probiotics, novel postbiotics, and FMT/FVT remain experimental (Yang Q. et al., 2023). Antibiotic exposure during radiotherapy can disrupt ecosystems and blunt engraftment, making timing and baseline profiling critical for selecting patients and preserving efficacy (Poonacha et al., 2022).

### 3.4 Predictive biomarkers and precision models

#### 3.4.1 Clinical nomograms

In cervical cancer cohorts, clinical–dosimetric nomograms have been developed to predict acute RE risk. A 2025 study by Zhu et al. created a nomogram for predicting ≥Grade 2 acute RE in older cervical cancer patients, incorporating age, total radiotherapy dose, bowel dose–volume parameters, and baseline features such as body weight (Zhu et al., 2025). Predictive performance was modest (AUC ~0.74 in training and validation), identifying advanced age and higher pelvic dose as predictors (Zhu et al., 2025). Such tools can support pre-treatment risk stratification and targeted prophylaxis.

#### 3.4.2 Radiomic and imaging models

Imaging-based models add dynamic prediction during treatment. Delta-radiomics from serial pelvic MRI in cervical cancer cohorts showed rectal-wall texture changes can identify developing radiation proctitis with high accuracy (AUC >0.90), suggesting microstructural remodeling precedes overt toxicity (Deng et al., 2025; Xue et al., 2025). Deep-learning models trained on serial CT/MRI can grade inflammation and predict severe enteritis, supporting real-time surveillance (Elhaminia et al., 2025). These approaches align with adaptive care: early imaging signals could trigger intensified symptom control, hydration, and, when needed, brief treatment pauses to prevent escalation.

#### 3.4.3 Microbiome–metabolome biomarkers

Multi-omics offers high-resolution susceptibility profiling. A prospective study integrating baseline microbiome composition with fecal/blood metabolites and select host inflammatory markers predicted severe acute RE in cervical cancer patients with AUC ~0.975; discriminators included SCFA-producing genera (e.g., *Faecalibacterium*) and metabolites such as phenylethylamine (Deng et al., 2025). Other candidates under investigation include baseline serum IL-22, longitudinal fecal calprotectin, and host genetic variants in DNA-repair or cytokine-related genes (e.g., OPN, PAI-1, KLF5, MTG16) that may modulate radiosensitivity or intervention response (Li et al., 2015; Wang J. et al., 2025).

#### 3.4.4 Standardization and clinical integration

Biomarker signatures require prospective validation and harmonization to reduce overfitting and platform effects; reproducibility depends on standardized sequencing/metabolomics pipelines and consistent sample timing. Clinically, tools could guide pre-treatment prophylaxis, mid-treatment escalation, and post-treatment surveillance for late toxicity. Risk models can also enrich high-risk patients in trials and support stratification, improving power while sparing low-risk patients unnecessary interventions.

## 4 Clinical translation in cervical cancer radiotherapy

### 4.1 Incidence and clinical features in cervical cancer patients

Cervical cancer is typically treated with pelvic external-beam radiotherapy (EBRT) to ~45–50 Gy delivered in 1.8–2 Gy fractions, followed by cervix-directed brachytherapy boosts, often combined with concurrent platinum-based chemotherapy (Mazeron et al., 2016; You et al., 2019). While this multimodal regimen is effective for tumor control, it inevitably exposes adjacent bowel. Small-bowel loops, sigmoid colon, rectum, and anal canal can all receive substantial dose, making radiation enteritis (RE) a frequent complication that can precipitate treatment interruptions, increase infection risk, and cause long-term quality-of-life impairment.

Acute RE develops during or shortly after radiotherapy in most patients. Mild–moderate gastrointestinal toxicity (Grade 1–2; intermittent diarrhea, cramping) is reported in up to ~80%, whereas severe events (Grade ≥3; persistent diarrhea requiring IV fluids or tube feeding) occur in ~20–30% (Ma et al., 2023; Zhu et al., 2025). Rates vary by technique (older 2D/3D plans vs contemporary IMRT/VMAT that better spare bowel) and patient factors such as age and comorbidities (Ma et al., 2023). Even with modern IMRT, one study in an older cervical cancer cohort reported 65% Grade ≥2 acute enteritis, underscoring heightened vulnerability with frailty and comorbidity (Zhu et al., 2025).

Chronic RE is less common but clinically important. Approximately 5–15% of survivors develop late gastrointestinal sequelae affecting daily life, typically emerging 6–24 months after treatment (occasionally years later) (Mazeron et al., 2016; Chater et al., 2019). Manifestations include chronic diarrhea (~5%), fecal urgency or incontinence, rectal bleeding from telangiectatic mucosal vessels (~5–10%), strictures causing partial bowel obstruction in a smaller subset, and rare fistulas (<2% in historical series, but high morbidity) (Mazeron et al., 2016; Chater et al., 2019). Histopathological subtypes—mixed, fibrotic, telangiectatic, and ulcerative—can all occur and may overlap within the same patient (Chater et al., 2019).

Several pattern–phenotype links are clinically relevant. Telangiectatic rectal lesions often drive recurrent bleeding and may coexist with fibrotic stenosis of the sigmoid colon (Takeuchi et al., 2012). Ulcerative necrosis can progress to fistula formation, particularly when high brachytherapy doses to the posterior vaginal fornix create rectal-wall hotspots (Mazeron et al., 2016). Rectovaginal fistulas are recognized late complications, especially in patients with prior pelvic inflammatory disease or very high cumulative brachytherapy dose to the rectovaginal septum (Yu et al., 2023).

A cervical cancer–specific nuance is brachytherapy’s steep dose gradient: the central target receives very high dose, and the anterior rectal wall can lie millimeters away. Consequently, highly localized injury—such as a single deep anterior-rectal ulcer or focal telangiectasia adjacent to the cervix—may occur even when overall pelvic constraints are respected (Mazeron et al., 2016; Deng et al., 2025). When identified, such focal lesions may be amenable to focal endoscopic therapy (argon plasma coagulation for telangiectasias; targeted steroid injection into an ulcer base).

Concurrent cisplatin, used as a radiosensitizer, may further sensitize intestinal mucosa and exacerbate acute symptoms (Ma et al., 2023). Cisplatin-related nausea, anorexia, and mucositis can contribute to malnutrition and delayed mucosal healing. In low-resource settings, baseline nutritional deficiencies, co-infections (e.g., HIV with impaired mucosal immunity), and limited access to supportive care add complexity. Differentiating radiation proctitis from recurrent tumor is also challenging because both can present with bleeding and strictures; however, biopsy of radiation-induced ulcers can heal poorly and may precipitate fistula, requiring cautious, multidisciplinary decision-making (Deng et al., 2025; Xue et al., 2025) (**Table 3**).

**Table 3:** Emerging therapeutic interventions for RE in cervical cancer radiotherapy.

Intervention	Mechanism	Evidence level	Preclinical evidence	Translational readiness	Potential benefits/challenges
Nrf2 Agonists *e.g., Formononetin, Swertiamarin*	Activate Nrf2/Keap1 to upregulate HO-1 and GPX4; reduce ROS, cGAS–STING activation, and toxic bile acids	Rodent (mouse models)	Restores intestinal barrier function in irradiated mice (12–21 Gy); reduces villus atrophy and ferroptosis	Preclinical	Potential prophylactic use in high-dose RT; requires isoform-specific Nrf2 activation to avoid protecting tumor cells
Engineered Probiotic Strains *e.g., modified E. coli Nissle variants, L. rhamnosus GG*	Overexpress SOD/CAT enzymes for ROS scavenging; restore SCFA (butyrate) production and indole metabolites; rebalance TH17/Treg responses	Rodent (mice/rats)	Single oral dose reduces apoptosis in abdominal-irradiation rodent models; restores gut microbiota composition in mice	Preclinical	Targets radiation-induced dysbiosis; could be combined with prebiotics (synbiotics) for chronic RE management. Challenge: ensuring probiotic engraftment and stability long-term
Mesenchymal Stem Cell (MSC) Therapies *e.g., adipose MSCs, MSC-derived exosomes, apoptotic vesicles*	Promote mitophagy and DNA repair (enhance HR/NHEJ); suppress TH17/IL-17A inflammation; stimulate intestinal stem-cell proliferation via RSPO1 secretion	Large Animal (dogs)	Improves histopathology in mouse and dog RE models; ~50% histological improvement observed in irradiated beagle study	Preclinical	Powerful immunomodulation for both acute and chronic phases; already in trial for other inflammatory conditions. Challenge: delivery and potential off-target effects (especially in genetically modified MSC products)
Ferroptosis Inhibitors *e.g., PPARγ agonists, Corilagin, Aloperine*	Shift glucose metabolism from glycolysis to hexosamine pathway; inhibit GAPDH acetylation; restore GSH balance by targeting AKR1C2	Rodent (cells & mice)	Alleviate inflammatory cell death (pyroptosis) in human IEC cell cultures and in mice; reduce Fe2+ accumulation and lipid peroxidation in irradiated intestine	Preclinical	Unique mechanism (glycosylation-dependent iron modulation); could be combined with RT to sensitize tumors while protecting normal tissue. Need to repurpose existing drugs (e.g., PPARγ agonists) with safety monitoring
Fecal Microbiota/Virome Transplant *FMT/FVT*	Enriches beneficial genera (*Roseburia*, *Akkermansia*); suppresses aberrant RIG-I/Notch signaling; restores microbial indole metabolite pathways	Rodent (mice/rats)	Ameliorates intestinal stem cell damage in irradiated mice; reduces gut inflammation via restored anti-inflammatory microbial metabolites	Preclinical	Leverages an existing clinical procedure (FMT) to rebalance microbiota. Promising for refractory cases, but donor variability and uncertain long-term engraftment pose challenges. Human trials (Phase I/II) are needed to establish efficacy in RE
Compound *Kushen* Injection *(Traditional herbal extract)*	Activates cannabinoid receptor 1 (CB1) signaling; downregulates radiation-induced inflammatory cytokines and fibrosis pathways	Rodent (rats)	Improves intestinal injury indices in rat models of chronic radiation enteritis (less mucosal inflammation and fibrosis)	Repurposed (adjunct cancer therapy)	Multi-component TCM remedy already used as an adjunct in cancer care; offers broad anti-inflammatory effects. Requires standardized formulation and controlled clinical trials to validate efficacy for radiation enteritis

**Table 3** presents emerging and investigational therapeutic interventions for radiation enteritis, detailing their underlying mechanisms, levels of experimental and clinical evidence, translational readiness, and associated benefits and challenges.

### 4.2 Risk prediction and personalized mitigation in cervical cancer cohorts

Several tools have been validated in cervical cancer radiotherapy cohorts and can guide risk-adapted mitigation. Using the nomogram by Zhu et al. as an example, clinicians can estimate an elderly patient’s pre-treatment probability of ≥Grade 2 acute RE (Zhu et al., 2025). If projected risk is high, preventive measures can begin before radiotherapy, including intensive dietary counseling to maintain adequate nutrition and, where feasible, support microbiome resilience while avoiding irritating or gas-producing foods (Venkidesh et al., 2023; Jo et al., 2024). Prophylactic pharmacotherapy can be considered; bile-acid sequestrants (cholestyramine or colestipol) may be started early to bind luminal bile acids that can irritate irradiated mucosa (Stryker et al., 1983). In selected settings, emerging bowel-protection techniques—such as temporary rectovaginal hydrogel spacers placed during brachytherapy to physically displace the rectum from high-dose regions—offer a mechanistic, anatomy-based mitigation strategy (Damato et al., 2017; Muramoto et al., 2023).

During radiotherapy, radiomics and biomarker tools provide opportunities for mid-course adaptation. If delta-radiomics flags subclinical injury on weekly imaging (cone-beam CT or MRI within adaptive workflows), supportive care can be escalated promptly: early anti-diarrheal initiation and dose escalation (loperamide or diphenoxylate at the first loose stools), rapid IV fluids and electrolyte repletion at early dehydration signals, and, if moderate toxicity develops, a short radiotherapy break to permit mucosal recovery without materially compromising tumor control (Wu et al., 2023; Ma CY. et al., 2025). During interruptions, nutritional support can be intensified, and adherence to adjunctive measures (e.g., probiotics or glutamine when used) can be reinforced, as fatigue and GI upset often drive lapses (Liu et al., 2017).

Biomarker surveillance can complement imaging for early detection of escalating injury. Weekly rises in fecal calprotectin or lactoferrin (markers of intestinal inflammation and neutrophil activity) may precede overt symptoms and justify earlier topical anti-inflammatory therapy (mesalamine suppositories) or short courses of corticosteroid enemas before severe bleeding or diarrhea occurs (Hille et al., 2008; Wu et al., 2018). If validated, mid-treatment increases in circulating IL-6 that anticipate impending severe enteritis could prompt targeted intervention (e.g., IL-6 inhibition) or, at minimum, closer observation and earlier escalation of supportive care (Bell et al., 2019).

Risk models also strengthen clinical trial design and patient triage. Trials of preventive therapies (novel probiotics, anti-TNF strategies) can enrich enrollment for high-risk patients—the subgroup most likely to benefit—thereby improving statistical power, while sparing low-risk patients from unnecessary medications or invasive procedures (Pineton de Chambrun et al., 2020; Jo et al., 2024). In routine practice, refined tools could support tiered care pathways: high-risk patients receive an “escalated supportive-care package” (scheduled medications, more frequent visits, dietary supplementation), whereas low-risk patients receive standard supportive care, concentrating resources where they are most needed (Tremblay et al., 2021).

### 4.3 Challenges and future directions

Despite progress, key barriers hinder translation of RE strategies in cervical cancer. Model–reality gaps remain substantial: many preclinical studies use single high-dose abdominal irradiation in rodents housed under specific-pathogen-free conditions with uniform microbiomes and genetics, whereas patients receive fractionated pelvic radiotherapy (~1.8 Gy daily over 5–6 weeks) plus brachytherapy boosts and often concurrent cisplatin (Mazeron et al., 2016; You et al., 2019). A one-time 13 Gy exposure and a multiweek clinical regimen generate fundamentally different tissue-response dynamics. Rodents also fail to reproduce key female-specific clinical context, including the hormonal milieu of premenopausal patients and the consequences of radiation-induced ovarian failure, which may alter mucosal biology and the gut–vaginal microbiome axis (Leibowitz et al., 2021; Zitter et al., 2023). Translational confidence will require fractionated and combined-modality models, and in some contexts large-animal models that better approximate human GI anatomy, despite higher cost and complexity.

Microbiota generalizability further limits translation. Baseline gut community structure varies across populations, diets, and environments; taxa linked to protection in one cohort (e.g., Akkermansia in 3HB-associated radioprotection) may be abundant in some regions and rare in others, limiting portability of biomarkers and probiotics (Song and Shin, 2022; Achasova et al., 2025). Even when protective strains are identified, colonization depends on diet (notably fiber), antibiotics, proton-pump inhibitors, and other exposures, producing variable outcomes (Hu L. et al., 2025). Population-specific validation and context-aware personalization—potentially using defined consortia designed for robust engraftment across gut environments—may be necessary.

Evidence quality is another constraint. Many microbiome and metabolomic studies enroll fewer than 50 patients, increasing overfitting and confounding (e.g., “protective” taxa acting as proxies for antibiotic nonuse), and batch effects in sequencing and metabolomics impede cross-study comparability (Li et al., 2023; Yu et al., 2024). Progress requires standardized sampling schedules (before, during, after radiotherapy), harmonized processing and analytic pipelines, and larger multi-center cohorts and pooled registries to enable external validation.

Regulatory and safety hurdles are substantial for live interventions. Engineered probiotics and fecal microbiota transplantation (FMT) are regulated as live biotherapeutic products and require stringent manufacturing, quality control, and phased safety evaluation (Pan et al., 2025; Rodriguez et al., 2025). In immunocompromised subsets (including patients with HIV), risks include infection and transfer of undesirable genes, necessitating careful donor/strain selection, monitoring, and clear eradication options (Wardill et al., 2019).

Patient heterogeneity and technique variability complicate implementation. Cervical cancer affects patients across ages 20s–80s with diverse comorbidities; diabetes, peripheral vascular disease, and smoking can impair mucosal healing and modify RE risk (Su et al., 2025). Radiotherapy technique varies (older 2D plans, IMRT/VMAT, proton therapy), yielding different bowel dose distributions, and anatomy (e.g., low-lying small-bowel loops) can increase toxicity risk even with IMRT (Portelance et al., 2001). Integrating granular dosimetry with biological predictors will likely provide the most accurate stratification.

Long-term monitoring remains inconsistent. Chronic RE may manifest years after treatment, yet survivors—particularly in low-resource regions—may be lost to follow-up or lack access to specialty care; effective options such as hyperbaric oxygen therapy for chronic radiation proctitis may be unavailable or unaffordable (Stout et al., 2024; Cooper and Hanley, 2025). Timing and sequencing of emerging therapies also remain unresolved: acute and chronic RE likely require different windows (e.g., microbiome support before and during radiotherapy vs reserving cellular therapies for severe injury) (Wang et al., 2021). Critically, microbiome-directed interventions must be evaluated for potential effects on tumor response to radiotherapy and concurrent immunotherapies; gut-targeted delivery (e.g., rectal administration of metabolites or microbes) may mitigate systemic immune perturbation (Yang et al., 2024; Lei et al., 2025).

Effective management is inherently multidisciplinary, requiring coordination among radiation oncology, gastroenterology, nutrition, and surgery. Distinguishing radiation injury from recurrence often needs integrated assessment; biopsy of radiation ulcers can impair healing and precipitate fistula, sometimes favoring empiric management (Dormand et al., 2005; Yu et al., 2023). Severe strictures or fistulas may necessitate surgery, but operations on irradiated bowel carry high risk (Turina et al., 2008). Incorporating patient-reported outcomes into survivorship follow-up can detect chronic symptoms earlier and align endpoints with meaningful quality-of-life benefits.

Future Research Priorities: (i) develop standardized fractionated and chemo-radiation models incorporating diverse microbiota and, where feasible, large-animal systems; (ii) build multi-center registries linking dosimetry, treatment factors, comorbidities, microbiome profiles, and longitudinal outcomes; (iii) design biomarker-driven trials (e.g., FMT for severely dysbiotic baselines; IL-6–targeted strategies for high IL-6 during treatment); (iv) compare prophylactic interventions head-to-head and test rational combinations; and (v) expand patient-derived platforms (intestinal organoids, ex vivo cultures, gut-on-chip devices) to study human-specific mechanisms and accelerate precision testing of interventions.
